# Functional connectivity changes in obsessive-compulsive disorder following exposure and response prevention

**DOI:** 10.1038/s41398-026-04192-2

**Published:** 2026-07-11

**Authors:** Lise Skarstein Jakobsen, Olga Therese Ousdal, Bjarne Hansen, Gerd Kvale, Kristen Hagen, Chris Vriend, Odile A. van den Heuvel, Anders Lillevik Thorsen

**Affiliations:** 1 Department of Biomedicine, University of Bergen, Bergen, Norway; 2 Bergen Center for Brain Plasticity, Haukeland University Hospital, Bergen, Norway; 3 Department of Radiology, Haukeland University Hospital, Bergen, Norway; 4 Center for Crisis Psychology, University of Bergen, Bergen, Norway; 5 Department of Clinical and Biological Psychology, University of Bergen, Bergen, Norway; 6 Psychiatric Department, Hospital of Molde, Molde, Norway; 7 Department of Anatomy and Neurosciences and Department of Psychiatry, Amsterdam UMC, Vrije Universiteit Amsterdam, De Boelelaan, 1117 Netherlands; 8 Compulsivity, Impulsivity and Attention Program, Amsterdam Neuroscience, Amsterdam, Netherlands

**Keywords:** Neuroscience, Psychology

## Abstract

Obsessive-compulsive disorder (OCD) has been increasingly linked to alterations in functional connectivity (FC) across large-scale brain networks, particularly the default mode (DMN), salience (SN), and central executive network (CEN), known as the triple-networks. However, little is known about how connectivity patterns evolve following treatment. We examined both static and dynamic resting-state FC in 25 adults with OCD and 21 matched healthy controls (HCs) at baseline, one week, and three months following intensive exposure and response prevention (ERP) using the Bergen 4-Day Treatment (B4DT). Independent component analysis (ICA) and sliding-window approaches were used to evaluate network connectivity within and between the triple networks. At baseline, adults with OCD showed stronger static connectivity within the DMN and SN and spent more time in a dynamic connectivity state characterized by strong connectivity within the DMN and decoupling between the DMN and SN compared to controls. Following the 4-day intensive treatment, these abnormal patterns normalized and remained stable at the three-month follow-up. A higher number of transitions between states was associated with reductions in the severity of OCD, anxiety, and depressive symptoms. Additional correlation analyses indicated that higher baseline SN-CEN connectivity was associated with greater symptom severity, while post-treatment reductions in CEN and CEN-SN static connectivity were linked to improvements in depressive symptoms. These findings suggest that effective ERP promotes a normalization of both static and dynamic connectivity within and between the triple networks. Thus, dynamic metrics, alongside static measures, may serve as clinically meaningful biomarkers of therapeutic response.

## Introduction

Obsessive-compulsive disorder (OCD) is characterized by intrusive, anxiety-provoking thoughts, images, or urges (obsessions), and repetitive, exaggerated mental or physical behaviors (compulsions), affecting 2–3% of the population [1]. The disorder is associated with an impairing course if left untreated, for which cognitive behavioral therapy (CBT), including exposure and response prevention (ERP) [2, 3], and selective serotonin reuptake inhibitors (SSRIs) constitute established first-line treatment options [1]. Although the pathophysiology of OCD remains incompletely understood, prevailing neurobiological models have implicated dysfunction within and between cortico-striato-thalamo-cortical (CSTC) brain circuits [1, 4–7]. More recently, evidence from resting-state functional magnetic resonance imaging (rs-fMRI) studies suggests that disruptions in the organization and functional communication of large-scale brain networks, beyond the traditional CSTC loops, may contribute to the underlying neurobiology of OCD [8–10]. A meta-analysis by Gürsel and colleagues [11] points to several alterations in functional connectivity (FC) within and between the default mode network (DMN), the salience network (SN), and the central executive network (CEN). The alterations within this triple-network model of Menon [12] have been linked to hallmark OCD symptoms [11], including maladaptive self-focus [13], cognitive inflexibility [11, 14], and executive dysfunction [14, 15]. Moreover, functional alterations between hubs of the DMN, CEN, and SN have been proposed as potential markers of treatment response in both pediatric [16] and adult individuals with OCD [17–19].

While most large-scale brain network studies have evaluated static FC [8], recent work suggests that dynamic FC is a more sensitive index of functional brain organization [8, 20–22]. In contrast to static FC, which measures the average interregional patterns in FC during the entire scan duration, dynamic FC includes the present dynamic and time-varying information in the rs-fMRI data. Interindividual alterations of dynamic FC during rest have been demonstrated in several psychiatric and neurological conditions, including major depressive disorder (MDD) [23], attention-deficit/hyperactivity disorder (ADHD) [24], autism spectrum disorder (ASD) [25], schizophrenia, and bipolar disorder [26]. The consideration of altered dynamic FC in patients with OCD is, however, limited, and the existing studies of resting-state FC are mixed, both in findings and methodologies [8, 9, 11]. We previously applied graph theoretical measures for dynamic FC to uncover decreased flexibility of the subgenual anterior cingulate in adults with OCD relative to HCs one week after intensive concentrated ERP [27]. This suggested that ERP may lead to a more independent and stable network topology. Related network-level changes have also been reported following pharmacological treatment, including SSRI-related alterations in frontoparietal FC and connectivity-degree in ventral frontal regions [28]. However, studies applying other dynamic FC methods and longer follow-up times are needed to understand if, and how, treatment affects the functional brain organization.

Recurrent time-varying connectivity during resting-state fMRI can be characterized as distinct brain states [29–32]. Two commonly used metrics to describe the interindividual dynamic properties of the dynamic states are the fraction of time, which measures the time spent in a brain state, and the number of transitions, which characterizes the frequency of transitions between the states. Recently, Li and colleagues [14] investigated both static FC and dynamic FC states in individuals with OCD and matched HCs in the largest sample to date within the triple-network model. They found that OCD patients exhibit stronger, static connectivity within the DMN and brain areas of the CEN. Both static and dynamic FC were weaker between the CEN and SN in OCD, whereas the dynamic FC state analyses also found weaker CEN-DMN connectivity in OCD compared to HC. This adds to the growing characterization of dynamic connectivity alterations in OCD presented in prior resting-state dynamic FC studies. Herein, stronger connectivity within the DMN in OCD has been reported by Gürsel et al [11] and Luo and colleagues [33], who also linked this to elevated anxiety levels. Luo et al [33] and Peng et al [34] found stronger dynamic FC within the SN in patients with OCD. Considering the CEN, Gürsel et al [11] found weakened CEN-connectivity to be positively correlated to OCD symptom levels, while Ding et al [35] identified weak dFC between the supplementary motor area and both the dorsolateral prefrontal cortex (DLPFC) in the CEN and the precuneus in the DMN in adults with OCD compared to HCs. Lastly, Liu et al [36] found that the number of transitions between states was higher in patients with OCD, and positively correlated with OCD symptom severity. Although limited in numbers, the dFC studies in OCD reveal tendencies of alterations within the triple networks, both supporting and contradicting current findings from static connectivity and related methodologies [9]. Accordingly, there is a need for further exploration, where treatment-associated changes in static and dynamic FC related to clinical outcomes could provide novel insight into connectivity patterns underlying OCD symptoms.

To the best of our knowledge, no study has investigated whether dynamic FC states change following successful treatment in OCD. In this study, we examined both static and dynamic FC using the triple-network model in OCD patients directly before-, three days after-, and three months following a concentrated ERP format, relative to matched HCs undergoing the same longitudinal scanning protocol. Accordingly, this longitudinal case-control design allows characterization of connectivity changes over time associated with clinical improvement; however, causal inference is limited by the absence of an untreated OCD comparison group. By combining static and dynamic functional analyses with clinical measures before and after ERP, this study seeks to provide a more comprehensive understanding of the neurobiological mechanisms underlying treatment response and short- and long-term symptom change in OCD.

## Materials and methods

### Participants

Thirty-five OCD patients and 31 healthy controls (HC) participated in this study and were also included in a previous study using graph theoretical measures of resting-state FC [27]. The patient group was recruited from an OCD clinic at Haukeland University Hospital, Bergen, Norway, and the HC through advertisements. The inclusion criteria included OCD as the primary diagnosis, aged 18 years or older, a minimum Yale-Brown Obsessive Compulsive Scale (Y-BOCS) score of 16, and fluency in Norwegian. Exclusion criteria for patients were symptoms primarily associated with hoarding, ongoing substance abuse, lifetime bipolar disorder or psychosis, suicidal ideation, intellectual disability, or being unwilling to refrain from alcohol or benzodiazepines during the two days of exposure treatment. HCs had no lifetime history of any psychiatric or severe somatic disorder and were matched according to sex, handedness, age, and education. All participants had to be without MRI contraindications and major neurological illnesses. The study was conducted in accordance with the Declaration of Helsinki and approved by the Regional Ethics Committee for South-Eastern Norway (2015/936). All participants gave informed written consent before participating in the study.

### Clinical measures

The Structured Clinical Interview for DSM-IV (SCID) was used to diagnose OCD and lifetime mental health disorders in patients and to rule out any current or lifetime disorders in healthy control subjects. Severity measures of OCD were assessed using the Yale-Brown Obsessive-Compulsive Scale (Y-BOCS) [37] at baseline, directly after treatment, and at a three-month follow-up. Remission after treatment was defined as a Y-BOCS score ≤ 12, and clinically meaningful improvement was defined as a minimum of 35% decrease in the Y-BOCS score based on international consensus criteria [38]. At every time point, all participants completed the Obsessive Compulsive Inventory-Revised (OCI-R), [39] a self-report measure of OCD symptom severity across six dimensions: washing, checking, ordering, obsessing, hoarding, and neutralizing. Additionally, the Patient Health Questionnaire (PHQ-9) [40] and the Generalized Anxiety Disorder 7 (GAD-7) [41] were administered to assess depressive and anxiety symptoms, respectively. A trained clinical psychologist performed the DSM-IV and Y-BOCS, and the patients were evaluated by an external rater not involved in the treatment during the period 2015-2018.

### Treatment

Individuals with OCD underwent the Bergen-4-Day Treatment (B4DT), a psychological treatment format implementing concentrated ERP in groups of 3-6 OCD patients over four consecutive days with a 1:1 ratio between patients and therapists. Effectiveness studies have shown that ~90% of patients respond, while the remission rate of the treatment is ~75% [42, 43], supported by randomized control and replication studies [44]. The treatment response is largely maintained four years after treatment [45]. The first treatment day involves a group psychoeducation session and planning of individual exposure tasks. The following two days consist of eight hours of therapist-assisted ERP in relevant settings. Between the second and fourth day, the patients are instructed to perform individual exposure at home. The last treatment day consists of sessions focused on summarizing the treatment, preventing relapse, and planning further self-exposure for the following three weeks [43].

### Image acquisition

MRI scans were performed on a 3 T General Electric Discovery MR750 (GE Healthcare, Milwaukee, Wisconsin, USA) equipped with an eight-channel head coil. High-resolution T_1_-weighted images were acquired using a sagittal fast spoiled gradient recall sequence with 192 slices, isotropic voxel size = 1mm^3^, repetition time (TR) = 7 ms, echo time (TE) = 3 ms, flip angle = 12°, slice thickness = 1.0 mm, and field of view (FOV) = 256 × 256 mm. For resting-state functional MRI (rs-fMRI) data, a gradient-echo-planar imaging (EPI) sequence was used to obtain 160 T_2_^*^- weighted echo-planar volumes with 34 slices, voxel size = 3.44 × 3.44 × 3.00 mm^3^, TR = 1800 ms, TE = 30 ms, flip angle = 80°, slice thickness = 3.0 mm, slice gap = 0.3 mm, FOV = 220 × 220 mm, matrix size = 64 × 64, performed in a bottom-up interleaved order. The total scan duration was 5 min, including five dummy scans for magnetization stabilization. Participants were instructed to lie still with their eyes closed without falling asleep.

### Data preprocessing

Preprocessing was performed using Statistical Parametric Mapping 12 (SPM) software (Wellcome Department of Cognitive Neurology, London, UK, http://www.fil.ion.ucl.ac.uk/spm/) implemented in MATLAB (v. 2021). The functional images underwent slice-time correction and realignment and were co-registered with the baseline T_1_-weighted structural image. Subsequently, the functional images were normalized to the standard Montreal Neurological Institute (MNI) template using unified segmentation, resampled to a 3 mm isotropic voxel size, and spatially smoothed with a 5 mm full-width Gaussian kernel at half-maximum, following Allen et al [21].

### Quality control

To control for head motion, the root mean square (RMS) displacement across all volumes and the dice coefficient between the mean-, smoothed-, and normalized EPI across all subjects versus each subject were estimated from the motion parameter outputs acquired in SPM. The quality control exclusion criteria consisted of a mean RMS displacement exceeding 0.2 mm, and/or more than 20 individual volumes with RMS displacement greater than 0.25 mm [30], and a dice coefficient below 0.9 between the individual scan and group average in MNI space to evaluate spatial consistency [46].

### Group independent component analysis in gift

**Fig. 1:**
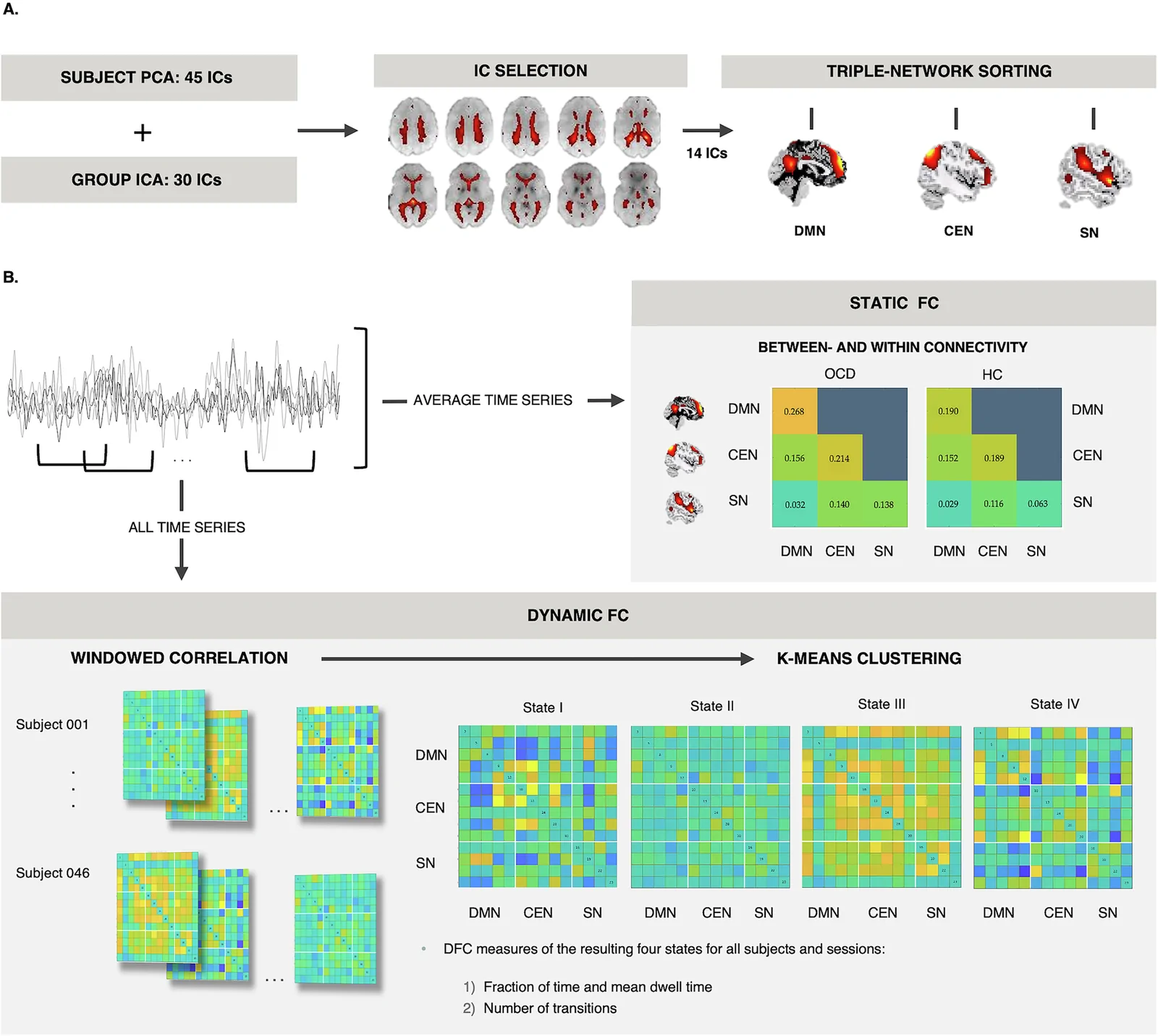
The analysis steps of the preprocessed and quality-controlled resting-state data. **A** Data reduction was performed using principal component analysis (PCA) on each subject, retaining 45 independent components (IC), and independent component analysis (ICA) for group reduction. The 30 ICs were evaluated based on spatial maps, time courses, and power spectra, and 14 ICs were sorted into the default mode network (DMN), central executive network (CEN), and salience network (SN). **B** Assessment of the triple network time series data. Static functional connectivity (FC) was assessed by pairwise connectivity correlations between and within the ICs of the DMN, CEN, and SN. For dynamic FC, the time series were partitioned into windows of correlation patterns, which were clustered into four representative states of recurring correlations.

The Group ICA of fMRI (GIFT v.4) Toolbox (http://mialab.mrn.org/software/gift/) was utilized to identify intrinsic functional resting-state networks through group independent component analysis (GICA) as illustrated in Fig. 1A. For group-level reduction, 30 independent components (ICs) were retained to identify functional parcellations aligned with the triple networks [47], while subject-level data were reduced using principal component analysis (PCA), retaining 45 principal components per subject. The ICs were estimated using the Infomax ICA algorithm [48] and repeated 20 times in ICASSO with bootstrap resampling [49]. Subject-specific spatial maps and time courses were reconstructed using the GICA back-reconstruction algorithm [50]. Spatial component maps were normalized using z-score scaling as proposed by Allen et al [21] to ensure comparability across participants. The spatial maps, time courses, and power spectra of the 30 ICs were classified as noise or signal using the criteria from Griffanti et al [51], and the sorted signal components were identified according to the methodology of Allen et al [30] and labeled following the triple-network model of Menon [12]. Resultingly, 14 signal components were sorted into CEN, SN, and DMN (Supplement 1). To reduce remaining noise sources and enhance network specificity, the time courses of the 14 ICs were post-processed in GIFT, including (1) detrending of linear, quadratic, and cubic trends, (2) despiking using 3D DESPIKE, (3) low-pass filtering with a high-frequency cut-off of 0.15 Hz, and (4) regressing out six motion realignment parameters.

### Static functional network connectivity analysis

Pearson correlations between the time series of each ICA-derived component of the DMN, SN, and CEN were computed and transformed to Fisher’s z-scores in MATLAB (v. 2023). For each MRI session, the average connectivity was calculated between (six variables) and within (three variables) the three networks for all participants (Fig. 1B).

### Dynamic functional network connectivity analysis

The dynamic Functional Network Connectivity (dFNC) toolbox within GIFT was used to investigate time-varying FC (Fig. 1B). The time series of the 14 ICs were segmented with a sliding window, using a window width of 44 s (22 TR) and a step size of 1 TR, shaped by a Gaussian function (σ = 3 TRs) [14, 21]. The resulting 139 windows were computed into 14 × 14 pairwise FC matrices using Pearson’s correlation, which were transformed to Fisher’s z-scores. To improve sparsity in the covariance matrices, an L1 norm penalty was implemented using the LASSO method of Friedman et al [52], reducing weaker connections in the covariance matrices. The analysis was repeated across 10 iterations, whereas the regularization parameter lambda (λ) was optimized separately for each participant. For classifying recurrent connectivity patterns across participants, a k-means clustering algorithm (Manhattan distance) was applied to the windowed covariance matrices and repeated 100 times with a maximum of 300 iterations per run, ensuring convergence [21]. The clustering procedure was repeated 20 times to test stability, and the cluster initialization was based on subject exemplars rather than random seed selection to improve consistency across runs. The optimal number of clusters was estimated using the elbow criterion of the cluster stability index, resulting in four FC states. To characterize the dynamic properties of FC, two key metrics were evaluated: (1) the fraction of time each participant spent in each state, and (2) the number of transitions between the four states.

### Statistical analysis

Statistical analyses were conducted using IBM SPSS Statistics 20.0. The normality of continuous variables was assessed with the Shapiro-Wilk test, and group comparisons at baseline were performed with independent samples t-tests and Mann-Whitney U tests. Fisher’s exact test was used to compare categorical variables between groups, and Pearson Correlation for bivariate correlation. Repeated measures ANOVA (RM-ANOVA) was employed to examine the main effects of group, time, and group-by-time interactions for clinical assessments, static correlations, and dynamic connectivity state metrics, accounting for dependencies within individuals. To ensure consistency, all analyses were limited to complete fMRI datasets, as the GIFT ICA toolbox does not accommodate missing data. Partial eta squared (ηp²) was used to report effect sizes. To address potential confounding variables, we repeated the analyses, including age, sex, and years of education as covariates. A significance threshold of p < 0.05 was set for all statistical tests. Both corrected and uncorrected results were reported, where the False Discovery Rate (FDR) was used for multiple comparisons.

## Results

### Demographic and clinical characteristics

Twenty-eight adults with OCD and 22 healthy controls (HC) completed the scan protocol at all time points. After quality control, one participant with OCD and one HC were excluded for excessive movement, and two participants with OCD were excluded for inadequate spatial normalization, resulting in a total sample of 46 subjects (25 OCD, 21 HC) for the final analysis, as GIFT requires complete fMRI data across all time points. Between-group comparability for age, sex, and years of education was reassessed in the final sample following attrition and quality control, demonstrating no significant differences in demographic variables. As expected, patients scored significantly higher on the PHQ-9, GAD-7, and OCI-R than HCs (Table 1).

**Table 1:** Baseline demographics, clinical characteristics, and longitudinal clinical assessment.

Characteristics	OCD	Healthy Controls	*p-*value
	*n* = 25	*n* = 21	
Age (mean years ± SD)	31.2 ± 9.3	34.1 ± 10.9	0.536^a^
Years of Education (mean years ± SD)	14.8 ± 2.2	14.4 ± 2.5	0.871^a^
Sex			
Female, n (%)	16 (64)	14(67)	1.000^b^
Handedness			
Right, n (%)	23 (92)	21 (100)	0.493^b^
PHQ-9 (mean ± SD)			
T0	10.0 ± 4.6 (n = 25)	2.5 ± 1.8 (n = 20)	<0.001^c^
T1	7.7 ± 5.2 (n = 21)	2.4 ± 2.0 (n = 20)	<0.001^c^
T2	7.9 ± 5.6 (n = 20)	2.0 ± 1.6 (n = 19)	<0.001^c^
GAD-7 (mean ± SD)			
T0	11.8 ± 5.1 (n = 25)	2.0 ± 2.6 (n = 20)	<0.001^c^
T1	7.7 ± 4.0 (n = 21)	1.8 ± 2.0 (n = 20)	<0.001^c^
T2	6.8 ± 4.6 (n = 21)	1.7 ± 2.0 (n = 19)	<0.001^c^
Y-BOCS (mean ± SD)			
T0	26.8 ± 4.1 (n = 25)	-	-
T1	9.8 ± 5.6 (n = 25)	-	-
T2	9.4 ± 6.1 (n = 24)	-	-
OCI-R (mean ± SD)			
T0	24.0 ± 9.5 (n = 25)	6.1 ± 7.6 (n = 20)	<0.001^c^
T1	11.4 ± 7.9 (n = 21)	5.3 ± 7.4 (n = 20)	0.016^c^
T2	12.4 ± 9.5 (n = 20)	4.6 ± 5.6 (n = 20)	0.004^c^
Comorbidities at T0, n (%)			
Anxiety^d^	18 (72)	-	-
Mood^e^	8 (32)	-	-
ADHD	1 (4)	-	-
Medication use at T0, n (%)			
SRI	6 (24)	0 (0)	0.011^c^
Methylphenidate	1 (4)	0 (0)	<0.001^c^

*ADHD* Attention Deficit Hyperactivity Disorder, *GAD-7* Generalized Anxiety Disorder 7, *OCD* Obsessive-Compulsive Disorder, *OCI-R* Obsessive-Compulsive Inventory-Revised, *PHQ-9* Patient Health Questionnaire 9, *SRI* Serotonin Reuptake Inhibitor, *T0* baseline, *T1* directly after treatment, *T2* three months after treatment, *Y-BOCS* Yale-Brown Obsessive-Compulsive Scale

^a^Mann-Whitney Test

^b^Fisher’s Exact Test

^c^Independent T-Test

^d^Social anxiety disorder and or generalized anxiety disorder, specific phobia, panic disorder, agoraphobia, post-traumatic stress disorder, hypochondriasis

^e^Major depressive disorder or dysthymia

### Treatment effectiveness

One week after treatment, patients with OCD showed a significant reduction from baseline on Y-BOCS (t(24) = 12.6, *p* < 0.001, d = 2.52), OCI-R (t(20) = 5.9, *p* < 0.001, d = 1.29), and GAD-7 (t(20) = 3.9, *p* < 0.001, d = 0.85), but not on PHQ-9 (t(20) = 1.94, *p* = 0.067, d = 0.42). Herein, 17 patients with OCD were in remission (68%), 23 of 25 patients clinically responded (92%), and two did not respond (8%). Three months after treatment, the initial reductions from baseline remained for the Y-BOCS (t(23) = 11.9, *p* < 0.001, d = 2.43), OCI-R (t(19) = 5.7, *p* < 0.001, d = 1.28) and GAD-7 scores (t(20) = 4.4, *p* < 0.001, d = 0.95). The PHQ-9 scores remained unchanged (t(19) = 1.4, *p* = 0.174, d = 0.32). Moreover, three additional patients were in remission, and 22 out of 24 patients were established as clinical responders. Accordingly, the reduction in symptom severity for OCD patients three months post-treatment revealed a total remission rate of 83% and a response rate of 92%. HCs showed no significant change in OCI-R, GAD-7, or PHQ-9 scores.

### Static network connectivity analyses

#### Baseline comparison

**Fig. 2:**
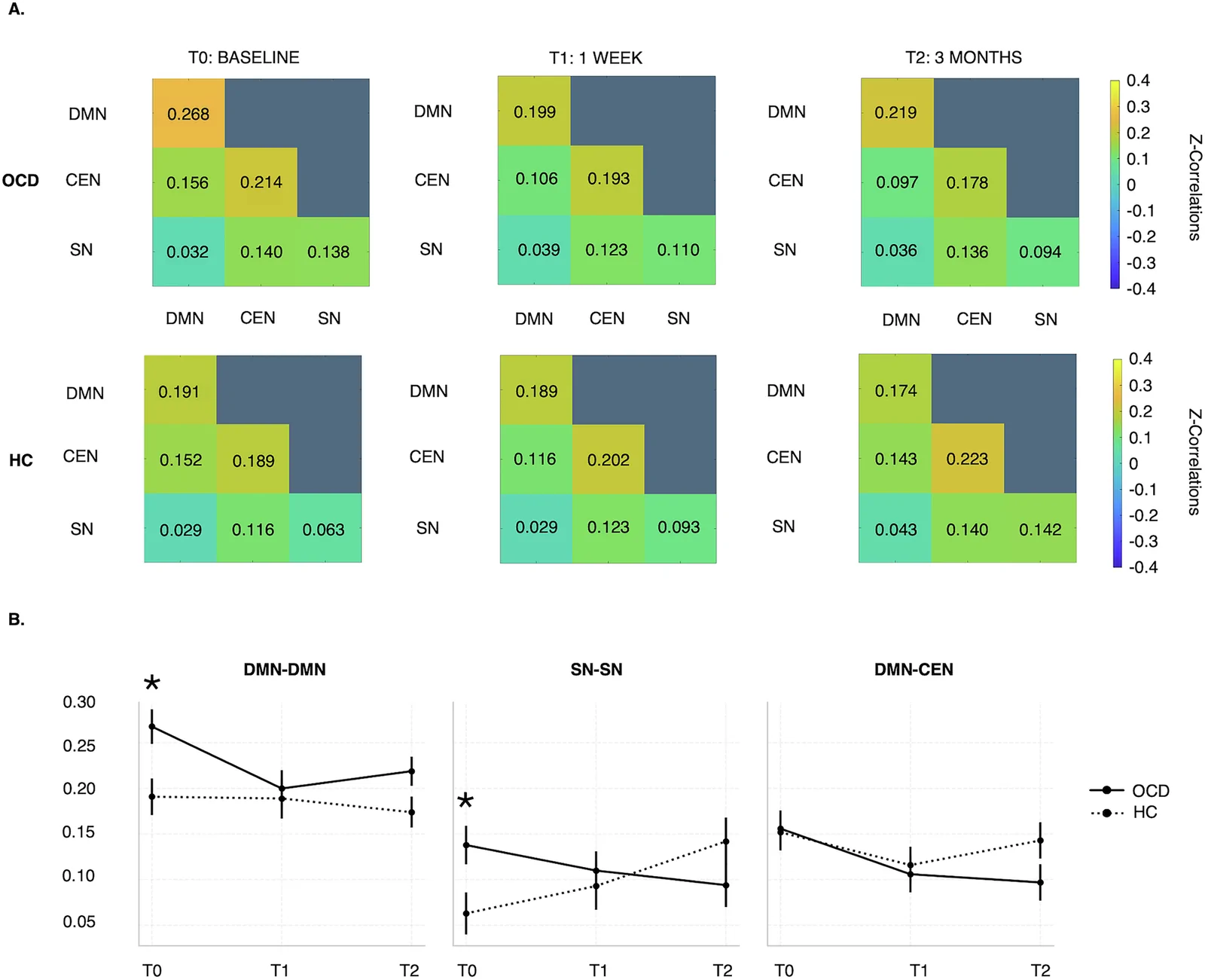
Static connectivity. **A** Correlation values of the connectivity within and between the default mode network (DMN), central executive network (CEN), and salience network (SN) at all time points. **B** Significant group-by-time changes in network connectivity between OCD and controls at all time points. *Significant difference between groups at baseline.

The OCD patients exhibited significantly higher connectivity within the DMN (t(44) = 2.799, p_FDR_ = 0.024, d = 0.828) and SN (t(44) = 2.437, p_FDR_ = 0.029, d = 0.721) compared to HCs (Fig. 2A+B). There were no significant group differences in connectivity within the CEN (t(44) = 0.792, p_FDR_ = 0.433, d = 0.234), nor for static between-network connectivity in any of the triple-network pairs at baseline.

#### Longitudinal analyses

Across all time points, the RM-ANOVA revealed a significant group-by-time interaction for connectivity within the SN (F(2,88) = 6.403, p_FDR_ = 0.017, ηp² = 0.127), indicating differential changes between the OCD group and HC over time. Herein, post-hoc comparison revealed a tendency towards decreased connectivity within the SN for the OCD group from baseline to three months post-treatment (t(24) = −2.260, p_uncorr_ = 0.033, d = −0.452). However, a comparatively significant increase was found in the HC group (t(20) = 1.596, p_FDR_ = 0.017, d = 0.705) (Fig. 2B).

A main effect of time was found in connectivity within the DMN (F(2,88) = 3.262, p_FDR_ = 0.043, ηp² = 0.069), characterized by a significant decrease within the OCD patients from baseline to one week after treatment (t(24) = 5.564, p_FDR_ = 0.017, d = 1.113), and from baseline to three months after treatment (t(24) = 2.735, p_FDR_ = 0.033, d = 0.530). At the same time, the HC group revealed no significant change over time (Fig. 2). We also found a significant main effect of time for connectivity between the DMN and CEN (F(2,88) = 5.073, p_FDR_ = 0.008, ηp² = 0.103), where individuals with OCD showed a significant decrease in connectivity both from baseline to one week after treatment (t(24) = 2.786, p_FDR_ = 0.033, d = 0.557), and baseline to three months after treatment (t(24) = 3.038, p_FDR_ = 0.017, d = 0.588). No significant change in network connectivity between the DMN and CEN occurred in the HC group over time; however, a decreasing trend from baseline to one-week follow-up, similar to the OCD group, occurred in the HC (t(20) = 2.075, *p* = 0.051, d = 0.453). There were no significant effects of time, group, or group-by-time interactions for connectivity within the CEN, between DMN and SN, or CEN and SN across all time points.

#### Correlations with clinical measures

Bivariate correlation tests showed a positive correlation between OCD symptom severity (Y-BOCS) and connectivity between the CEN and SN at baseline (r = 0.453, p_uncorr_ = 0.023). Also, a reduction in depressive symptoms (PHQ-9) in individuals with OCD from baseline to three months after treatment was correlated with decreased connectivity within CEN (r = 0.511, p_uncorr_ = 0.021) and decreased network-connectivity between the CN and SN (r = 0.528, p_uncorr_ = 0.017). No other correlations were found for baseline outcomes or changes between static connectivity and clinical measures.

### Characteristics of dynamic functional connectivity states

**Fig. 3:**
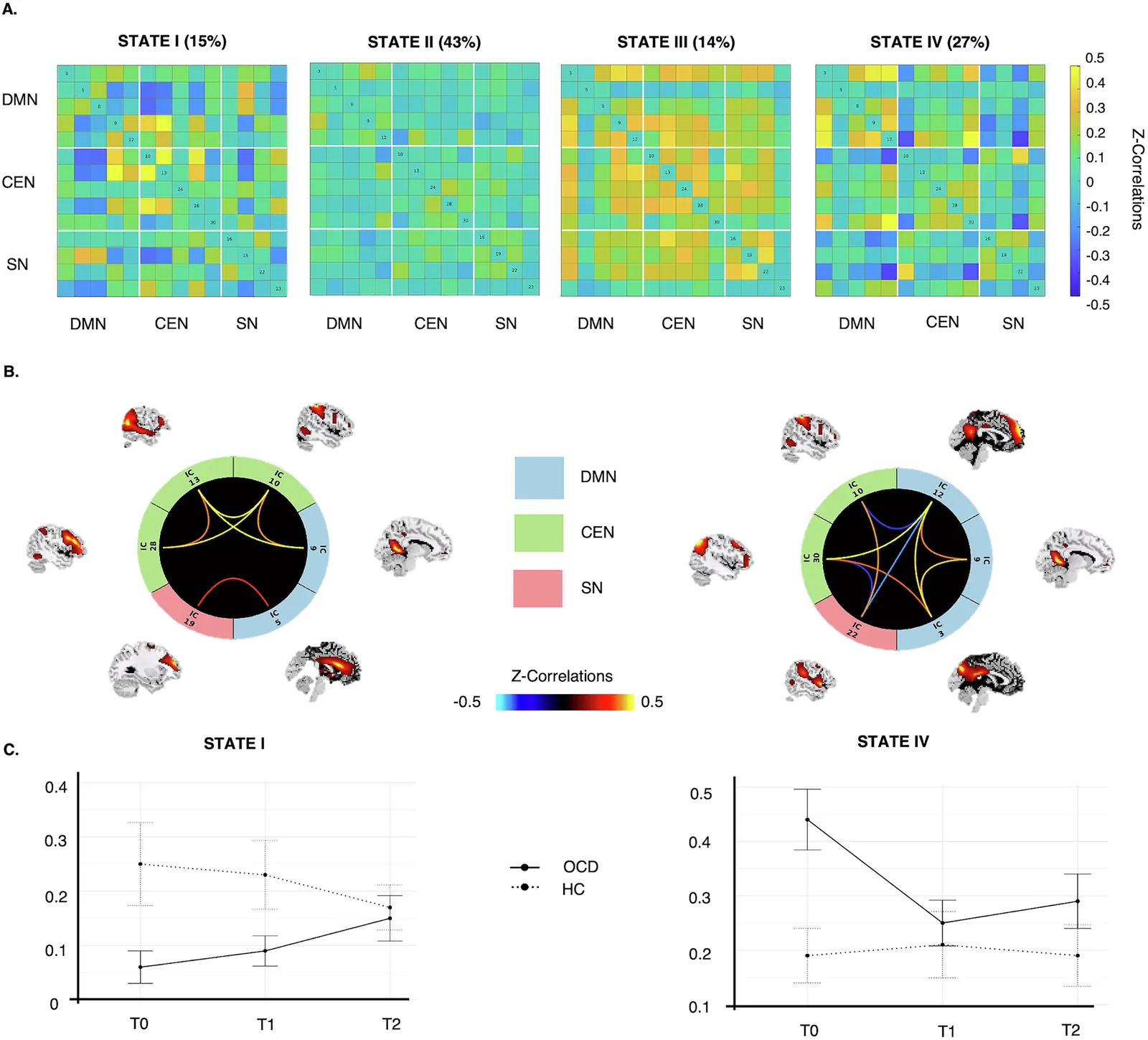
Dynamic Connectivity. **A** Correlation plots of recurring dynamic functional connectivity patterns between and within the 14 independent components sorted into the default mode network (DMN), salience network (SN), and central executive network (CEN) in all participants averaged across all time points. The percentages represent the overall time the participants spent in that state at all time points. **B** The correlation connectomes of state I and state IV, thresholded at a correlation minimum of ± 0.3 within and between the DMN, CEN, and SN. **C** The marginal means of the fraction of time in state I and state IV from baseline (T0), one week after treatment (T1), and three months after treatment (T2).

DFNC patterns across all individuals and time points were clustered into four states reflecting variable patterns of connectivity between and within the DMN, SN, and CEN (Fig. 3A). State I was predominantly characterized by positive within-CEN connectivity, and positive connectivity between the CEN and DMN. The SN exhibited generally weak within-network connectivity, whereas the between-connectivity with CEN and DMN revealed predominantly negative connectivity (Fig. 3B). State II was characterized by weak overall connectivity both between and within the three networks. In contrast, state III exhibited moderate positive connectivity within and between all networks. Lastly, state IV exhibited considerable positive connectivity within the DMN and negative connectivity between the DMN and SN. In addition, state IV exhibited both positive and moderately negative connectivity between the DMN and CEN, as well as mixed connectivity between the SN and CEN (Fig. 3B).

### Dynamic network connectivity analyses

#### Group differences at baseline

Before treatment, OCD patients spent significantly more time in state IV (t(44) = 3.394, p_FDR_ = 0.004, ηp² = 1.005) and less time in state I (t(44) = −2.385, p_FDR_ = 0.042, ηp² = −0.706) compared to HC (Table 2). There were no significant group differences in states II and III, or for the number of transitions at baseline.

**Table 2:** Baseline differences between OCD and HC in dynamic functional connectivity state metrics.

Baseline Characteristics	OCD (n = 25)	HC (n = 21)	t (d*f)*	*p*	Cohen’s D
Fraction of Time					
State I	0.064 ± 0.148	0.246 ± 0.349	−2.385 (44)	**0.021**^a^	−0.706
State II	0.378 ± 0.240	0.422 ± 0.304	−0.545 (44)	0.294	−0.161
State III	0.116 ± 0.165	0.145 ± 0.230	−0.502 (44)	0.618	−0.149
State IV	0.442 ± 0.276	0.186 ± 0.227	3.394 (44)	**0.001**^a^	1.005
Number of Transitions	4.32 ± 1.73	4.29 ± 1.66	0.058 (44)	0.954	0.017

*HC* healthy controls, *OCD* Obsessive-Compulsive Disorder

^a^Significant after FDR correction

#### Longitudinal analyses

RM-ANOVA showed a time-by-group interaction in fraction of time in state IV (F = 3.361, p_uncorr_ = 0.039, ηp² = 0.071), indicating a differential change between the OCD group and HC over time (Fig. 3C). Herein, post-hoc analyses revealed that the OCD patients showed a significant reduction in time spent in state IV from baseline to one week post-treatment (t(24) = 3.325, p_FDR_ = 0.006, ηp² = 0.665), and from baseline to three months after treatment(t(24) = 2.350, p_FDR_ = 0.027, ηp² = 0.470), whereas HC showed no significant change. There were no significant longitudinal changes in states I, II, and III, or the number of transitions. The inclusion of age, sex, and years of education as covariates did not alter these findings.

#### Correlation with clinical measures

Correlation analyses between the clinical scores of OCD severity (Y-BOCS, OCI-R), anxiety (GAD-7), and depression (PHQ-9) and the dynamic connectivity measures revealed no significant associations at baseline. However, three months after treatment, all clinical symptom measures were cross-sectionally negatively correlated with the number of transitions among connectivity states (Y-BOCS: r = −0.539, p_FDR_ = 0.014; OCI-R: r = −0.605, p_FDR_ = 0.005), anxiety (GAD-7: r = −0.449, p_FDR_ = 0.045), and depression (PHQ-9: r = −0.453, p_FDR_ = 0.014) (Table 3). Moreover, significant positive correlations were calculated three months post-treatment between time spent in state IV and OCD symptom severity (OCI-R: r = 0.488, p_uncorr_ = 0.029), and anxiety (GAD-7: r = 0.475, p_uncorr_ = 0.029), moderately associating time spent in state IV with higher OCD severity and anxiety levels (Table 3).

**Table 3:** Correlations between clinical measures and dynamic functional connectivity metrics three months after treatment (T2) and pre-post-changes from baseline to three months after treatment (T0-T2).

	Fraction of Time State I		Number of Transitions		Fraction of Time State IV	
	*r*	p	r	p	r	p
**Y-BOCS**						
T0-T2	−0.019	0.928	−0.357	0.087	−0.104	0.630
T2	−0.128	0.550	−0.539	**0.007**^a1^	0.149	0.487
**OCI-R**						
T0-T2	−0.103	0.200	−0.078	0.744	−0.212	0.370
T2	−0.238	0.312	−0.605	**0.005**^a1^	0.488	**0.029**^a^
**GAD-7**						
T0-T2	−0.387	0.083	−0.373	0.096	−0.456	**0.038**^a^
T2	−0.198	0.388	−0.449	**0.041**^a^	0.475	**0.029**^a^
**PHQ-9**						
T0-T2	−0.189	0.424	−0.457	**0.043**^a^	−0.287	0.220
T2	−0.198	0.404	−0.453	**0.045**^a^	0.241	0.306

*GAD-7* Generalized Anxiety Disorder 7, *OCI-R* Obsessive Compulsive Inventory-Revised, *PHQ-9* Patient Health Questionnaire 9, *T0* baseline, *T2* three months after treatment, *Y-BOCS* Yale-Brown Obsessive Compulsive Scale.

^a^Significant; ^1^ = *p*-value survives FDR correction.

Correlation analyses from baseline to three months after treatment associated increased time spent in state IV with less improvement in generalized anxiety symptoms (GAD-7: r = 0.456, p_uncorr_ = 0.038) (Table 3). Moreover, a significant negative correlation was found between the change in number of transitions and improvement in depressive symptoms (PHQ-9: r = −0.457, p_uncorr_ = 0.043), indicating that an increased number of transitions is associated with fewer depressive symptoms (Table 3).

## Discussion

In this resting-state fMRI study, we investigated how functional connectivity within and between the DMN, SN, and CEN in adults with OCD changes after ERP compared to healthy, matched controls. By analyzing both static and dynamic FC before, directly after, and at three months follow-up after considerable clinical improvement following ERP, we have provided novel insight into the modifiability of FC related to clinical outcomes in OCD. For static connectivity, the adults with OCD exhibited higher connectivity within both the DMN and SN relative to HCs at baseline, which decreased following treatment and remained lower at the three-month follow-up. Consistently, our dynamic analyses revealed a sustained post-treatment reduction in the time spent in a state characterized by positive connectivity within the DMN and negative DMN-SN connectivity, resolving the baseline group differences. Finally, clinical correlations linked greater OCD symptoms with higher static connectivity between the SN and CEN at baseline, and a higher number of dynamic state transitions after treatment. Lower depressive symptoms were also related to a higher transition number, as well as decreased static connectivity post-treatment within the CEN and between the CEN and SN in individuals with OCD.

Our static findings of stronger connectivity within the DMN and SN in OCD align with several previous ICA-based studies [11, 14, 15], which have been associated with altered self-referential processing and salience attribution, respectively [12, 53]. Specifically, stronger connectivity within the DMN is thought to reflect persistent, intrusive thoughts and ruminative loops observed in OCD [8, 53]. Overactivity within the SN has been linked to heightened interoceptive sensitivity and aberrant salience attribution, related to the exaggerated urgency and emotional salience of intrusive thoughts [8, 13]. Beyond salience detection, the SN has been described as a dynamic switch between the DMN and the CEN [54–57]. As the CEN is associated with externally directed and cognitively demanding processes [15], this dynamic property of the SN is thought to regulate the transitions between internally and externally oriented cognitive states [54–57]. Current evidence suggests that altered SN dynamics disrupt this triple-network coordination, where persistent DMN dominance and/or reduced regulatory engagement of the CEN [11, 14, 15] contribute to disengagement from irrational internal thoughts and inability to correct internal concepts with external evidence [9]. This aligns with our observation of weaker FC within the SN and DMN in the absence of group-level differences in the CEN. The reported reduction in CEN-DMN connectivity within OCD post-treatment complements this pattern.

Regarding treatment-related changes in our static analyses, we found a significant reduction in connectivity within the DMN and SN in OCD patients after treatment, which remained after three months. The HCs revealed stable connectivity within the DMN at all time points, while the SN connectivity increased over time in HCs. For OCD, this transition from hyperconnectivity within the DMN and SN towards the connectivity levels of HCs after treatment might reveal a treatment-related reorganization of aberrant network engagement. However, the findings should be replicated in future studies where individuals with OCD are randomized to a treatment or a waitlist control group [58].

Our dynamic connectivity analyses revealed that OCD patients at baseline spent significantly more time in a state characterized by higher connectivity within the DMN and negative connectivity between the DMN and SN relative to HC (State IV). This pattern is suggestive of prolonged engagement in an internally focused state with limited salience regulation, consistent with both our static findings and prior studies of dynamic connectivity in OCD [33, 36]. Crucially, the proportion of time spent in this state was significantly reduced following treatment and remained stable after three months. Also, OCD patients at baseline spent less time in a more integrative state (state I), defined by positive connectivity within the CEN, both negative and positive connectivity between DMN and CEN, and negative connectivity within the SN, and between SN-DMN and SN-CEN. This aligns with prior studies identifying CEN alterations in OCD due to its central role in orchestrating top-down control over internally generated spontaneous thoughts and images [11, 14]. Consistently, a stronger negative correlation between the DMN and frontoparietal network has been associated with greater sustained attention [13], suggesting that increased engagement in this integrative state may reflect improved functional organization and regulatory capacity.

The correlations between FC and clinical outcomes offer further support for the relevance of triple-network dynamics in OCD. Although we observed no group differences in the number of transitions between dynamic states at baseline, cross-sectional analyses three months post-treatment showed that a higher number of transitions was associated with lower OCD, anxiety, and depressive symptoms. Additionally, spending more time in the DMN-dominated state (State IV) three months post-treatment correlated with higher residual OCD and anxiety symptoms. This directly supports the findings by Reggente [18], who identified DMN connectivity as the main predictor of CBT outcomes. Furthermore, higher SN-CEN connectivity at baseline was associated with greater OCD severity in our sample. This pattern was also observed by Shi et al [19], who reported that higher connectivity between the anterior insula in SN and lateral prefrontal cortex (PFC) in CEN predicted poorer response to ERP. Although emphasizing baseline predictors, their interpretation supports the idea that excessive salience-driven executive coupling may reflect a maladaptive trait that interferes with cognitive flexibility and symptom improvement. Finally, greater reductions in depressive symptoms were associated with decreased static connectivity within the CEN and between the CEN and SN. This supports the task-based findings by Becker et al [59], who linked ERP-induced reductions in depressive symptoms in adolescents with OCD to decreased FC between CEN and DMN.

Findings from resting-state fMRI studies of SSRI treatment, the established first-line pharmacological intervention for OCD, may inform the interpretation of treatment-related network changes. Within this framework, pharmacological treatment effects in OCD have been described primarily at the level of large-scale intrinsic FC, using graph-theoretical measures. Specifically, Shin et al [28] observed post-treatment network reorganization following a 16-week SSRI intervention, including increased clustering coefficient and small-worldness, increased nodal degree in frontoparietal regions, and connectivity-degree changes in ventral frontal regions related to symptom improvement. Although methodologically distinct, these findings support a network-based view of OCD in which effective treatment is accompanied by reorganization of large-scale brain networks.

Our study combines data-driven ICA with static and dynamic connectivity analyses across multiple time points, where the inclusion of HCs at all time points enhances the interpretability of change trajectories, and substantial clinical improvement observed following ERP facilitates a solid premise for detecting treatment-associated changes in connectivity. However, several limitations must be considered. The modest sample size, together with the absence of an untreated OCD comparison group, limits statistical power and the ability to disentangle treatment effects from confounders or the natural course of OCD, respectively. In particular, a subset of 7/25 patients (28%) received concurrent pharmacological treatment, where associated influences cannot be excluded as contributing to the observed connectivity changes. Moreover, while sliding-window–based dynamic approaches are widely used, their reliability and interpretability remain an area of ongoing methodological development and validation [32]. In the present study, multiple steps were taken to mitigate known sources of instability, including stringent motion criteria, ICA-based denoising, despiking, temporal filtering, and stability-optimized clustering procedures. Nevertheless, dynamic connectivity metrics provide an indirect characterization of time-varying functional organization, and continued methodological development and replication are warranted.

In conclusion, our findings support a triple-network model of OCD, where exposure-based treatment not only alleviates symptoms but may also reconfigure FC large-scale brain networks as observed both in static and dynamic analyses. The observed reductions in FC within the DMN and SN, along with decreased engagement in a state dominated by the DMN, suggest that ERP may facilitate rapid normalization of maladaptive network dynamics. Connectivity patterns involving SN-CEN coupling and DMN-dominant states may hold promise as informative markers of treatment response and targets for future interventions. These results underscore the potential of network-based markers to inform precision approaches in OCD treatment and advance our understanding of large-scale brain, time-varying dynamics in mental disorders.

## Supplementary information

Supplement 1

## Data Availability

The data presented in this study are not available due to privacy restrictions.
